# Physical Health Decline After Chemotherapy or Endocrine Therapy in Breast Cancer Survivors

**DOI:** 10.1001/jamanetworkopen.2024.62365

**Published:** 2025-02-28

**Authors:** Clara Bodelon, Matthew Masters, Den E Bloodworth, Peter J. Briggs, Erika Rees-Punia, Lauren E. McCullough, Alpa V. Patel, Lauren R. Teras

**Affiliations:** 1 Department of Population Science, American Cancer Society, Atlanta, Georgia; 2 Emory University Rollins School of Public Health, Emory University, Atlanta, Georgia

## Abstract

**Importance:**

Women with a history of breast cancer (BC) experience greater physical health decline compared with age-matched women without cancer. However, whether this decline differs in patients who received chemotherapy and endocrine therapy is not well understood.

**Objective:**

To investigate physical health decline in BC survivors who received chemotherapy or endocrine therapy compared with age-matched women without cancer.

**Design, Setting, and Participants:**

This prospective cohort study was conducted as part of the Cancer Prevention Study-3, a prospective US cohort study that enrolled participants in 35 states, the District of Columbia, and Puerto Rico between 2006 and 2013. Follow-up for this analysis was through April 1, 2020. Statistical analyses were conducted between May 2023 and December 2024. Female participants diagnosed with nonmetastatic BC who returned a survey at least 90 days after their diagnosis were matched on age and year of survey return with up to 5 women without cancer.

**Exposure:**

Cancer status and treatment information.

**Main Outcomes and Measures:**

Outcomes of interest were measures of physical health, assessed using the Patient-Reported Outcomes Measurement Information System Global Health Scale instrument. Linear regression was used to estimate associations (β) and 95% CIs of treatment with physical health.

**Results:**

This analysis included 2566 individuals diagnosed with BC and 12 826 age-matched women without cancer. Median (IQR) age at diagnosis was 56.3 (49.9-61.9) years. Of women with BC, 1223 (47.7%) received endocrine therapy, 276 (10.8%) received chemotherapy, and 634 (24.7%) received both. Compared with women without cancer, there was a greater physical health decline within 2 years of diagnosis for BC survivors receiving endocrine therapy (β = −1.12; 95% CI, −1.64 to −0.60), chemotherapy (β = −3.13; 95% CI, −4.19 to −2.07), or both (β = −3.26; 95% CI, −3.97 to −2.55). The decline among endocrine therapy users was restricted to women receiving aromatase inhibitors. More than 2 years after diagnosis, the decline was only observed in women who received chemotherapy.

**Conclusions and Relevance:**

In this cohort study of 15 392 BC survivors and age-matched women without cancer, BC survivors who received chemotherapy had a long-lasting physical health decline, unlike survivors who received endocrine therapy without chemotherapy. Further studies are needed to confirm these results and to better understand the health consequences of these treatments.

## Key Points

**Question:**

Does physical health in breast cancer survivors who receive chemotherapy or endocrine therapy decline compared with age-matched women without cancer?

**Findings:**

In this cohort study of 15 392 women, breast cancer survivors receiving chemotherapy, endocrine therapy, or both had significant decline in physical health compared with age-matched controls during the first 2 years after the diagnosis. After 2 years, physical health decline was only observed in breast cancer survivors who received chemotherapy.

**Meaning:**

These results suggest that breast cancer survivors receiving chemotherapy had long-lasting physical health declines, unlike those who received endocrine therapy without chemotherapy.

## Teaser

This cohort study examines physical health measures in association with chemotherapy or endocrine therapy in women with breast cancer compared with age-matched women without cancer.

## Introduction

The number of women with a history of breast cancer (BC) continues to increase in the United States, and it is estimated to be more than 5 million by 2030.^1^ With advances in early detection and treatment, the 5-year relative survival for early-stage BC is greater than 90%.^2^ Because of the longer survival, patients with BC experience a plethora of long-term and late health effects, which are hypothesized to be the consequences of the cancer and its treatment.^3,4,5^ In particular, BC survivors are at risk of earlier onset and higher incidence of chronic health conditions compared with similarly aged individuals without cancer,^6^ which is thought to represent accelerate rates of aging. A well-studied aging phenotype in BC survivors is the decline in physical health.^7,8,9,10,11^ It constitutes a major cause of morbidity in this population. Understanding factors related to the physical health decline in BC survivors could lead to interventions to improve their health outcomes.^12^

BC is a heterogenous disease. Guideline recommendations to treat BC differ according to tumor characteristics and include various combinations of surgery, radiation, chemotherapy, and endocrine therapy. Most BCs (68%) are hormone receptor positive (ie, estrogen or progesterone receptor positive) and human epidermal growth factor receptor 2 negative.^13^ Since the early 2000s, treatment recommendations for these tumors have included endocrine therapy for at least 5 years.^14^ Radiation and chemotherapy therapy are cytotoxic therapies that induce DNA damage or interfere with DNA synthesis and replication leading to tumor cell death.^15^ However, these therapies also affect healthy tissues and cells,^16,17^ which may cause the accelerated aging effects observed in patients with cancer. In contrast, endocrine therapy acts by either suppressing transcription of estrogen-responsive genes or enzymatically inhibiting the production of estrogens.^18^ Therefore, the aging effects of endocrine therapy may differ from the effects of other treatment modalities. Many studies of physical health in BC survivors have not included treatment as part of their analysis^19,20^ or have focused only on the effects of chemotherapy or radiation.^11,21^

To address this gap, we examined physical health according to the treatment received in a large, contemporary cohort of women diagnosed with BC. We compared their physical health before and at 3 intervals after their BC diagnosis to age-matched women without cancer who provide a reference measure of physical health during normal aging.

## Methods

This cohort study was approved by the institutional review board of Emory University and those of participating registries as required. All participants provided informed consent. This study was conducted in accordance with the Strengthening the Reporting of Observational Studies in Epidemiology (STROBE) reporting guideline for cohort studies.

### Study Population

This analysis was conducted within the American Cancer Society Cancer Prevention Study 3 (CPS-3), a prospective cohort study that enrolled participants in 35 states, the District of Columbia, and Puerto Rico between 2006 and 2013.^22^ Eligibility criteria included being aged 30 to 65 years and no personal history of cancer other than nonmelanoma skin cancer. Enrollees provided a signed consent form and returned a baseline survey and triennial follow-up surveys beginning in 2015. Race and ethnicity were self-reported at enrollment, with 1 question asking about being Hispanic or Latino ethnicity and another about race with the following response categories: American Indian or Alaskan Native, Asian, Black or African American, Native Hawaiian or Pacific Islander, White, and other (with the option of a write-in text box). Participants were able to select multiple categories. Surveys were available in English and Spanish to facilitate the enrollment of Hispanic men and women. Race and ethnicity were included to describe the study population. Participants were linked to state cancer registries to identify all reportable cancer diagnoses.

Women diagnosed with a first primary incident BC were included in this analysis if they returned a survey at least 90 days after their diagnosis through April 1, 2020, the end of follow-up for this analysis (eFigure 1 in Supplement 1). Information collected from the baseline (completion period: 2006-2013), 2015 (completion period: 2015-2017), and 2018 (completion period: 2018-2020) surveys ware included in this analysis. We excluded 38 women who had a distant cancer, 64 women who did not undergo breast surgery if they were diagnosed with invasive disease, 165 women whose only postdiagnosis survey was within 90 days of their diagnosis, and 118 women who had any additional cancer diagnosis before returning any postdiagnosis survey. If they were diagnosed with an additional cancer during follow up (39 participants), only data from surveys prior to this additional diagnosis were included in the analysis. Up to 5 women without cancer were matched with BC survivors on age (±2 years) and year of survey returns (±1 year). To be included in the matched group, women had to be alive, in active follow-up, and cancer-free at the time of the postdiagnosis survey return of their matched case.^23^ If they developed a cancer during follow-up (106 women), only data from surveys prior to developing this cancer were included in the analysis.

Of 2566 women with BC included in this analysis, 2161 (84.2%) returned all 3 surveys, 132 (5.1%) returned only the baseline and 2018 surveys, and 273 (10.6%) returned only the baseline and 2015 surveys. All 39 women with BC who had an additional cancer returned all 3 surveys, but only the baseline and 2015 data were included. Of 273 women who did not return the 2018 survey, 17 died before the 2018 survey was administered and 28 returned their 2015 survey 5 or more years after their diagnosis. There were 228 women (8.9%) who returned their 2015 survey within 2 years from their diagnosis but did not return the 2018 survey at least 2 years after their diagnosis.

### Physical Health Assessment

The Patient-Reported Outcomes Measurement Information System (PROMIS) Global Health version 1.2 instrument was used to assess physical health in the 2015 and 2018 surveys. PROMIS consists of 10 global health items, with 4 of them focusing on measuring physical health based on self-report physical health, physical function or ability to carry out everyday activities of daily living (ADL), fatigue, and pain.^24^ Raw responses scores to the 4 items were added, and the scoring tables from the Global Health scoring manual^25^ were used to convert the sum to standardized T-scores. Lower values of the score indicate worse physical health. The baseline survey only included the self-reported physical health–related item, and data from this survey could only be used for the analysis of the self-reported physical health item.

### Treatment Information

First-course treatment information was obtained from cancer registries and self-report and included receipt of surgery, chemotherapy, radiation therapy, and endocrine therapy. Of 2556 women with BC included in this analysis, 2506 (97.7%) had registry data and 1871 (72.9%) had self-reported data. There were 1812 women (70.6%) who had data from both sources. The agreement between registry and self-report in these women was 99.4% for surgery, 97.4% for chemotherapy, 90.5% for radiation, and 85.0% for endocrine therapy. Based on these high percentages, a woman was considered to have taken a specific cancer treatment if there was an indication from the registries or self-report that she had received that treatment. The types of endocrine therapy, tamoxifen or aromatase inhibitors (AIs), were self-reported. We validated self-reported types of endocrine therapy via medical record abstraction in 15% of patients with BC. The sensitivity of using tamoxifen, ie, the proportion of people using tamoxifen as abstracted from the medical records who reported using tamoxifen, was 90.2%, and the sensitivity for AIs was 87.0%.

### Statistical Analysis

Associations between treatment received and the PROMIS physical health score were computed using linear regression. Patterns of treatment were coded as a categorical variable according to the different combinations of the individual treatments received. Since the mean physical health scores were not statistically different in treatment combinations with and without radiation therapy, we combined both, resulting in cancer-free, neither endocrine therapy nor chemotherapy, endocrine therapy without chemotherapy, chemotherapy without endocrine therapy, and chemotherapy plus endocrine therapy treatment categories (eTable 1 in Supplement 2). Analyses were conducted at 4 time points relative to the diagnosis date (Figure). A woman could have at most 1 physical health assessment in each interval. If more than 1 assessment was available for a woman in an interval, the closest to the diagnosis was included in the analysis. This was the case for only 126 BC survivors and their matched controls. Models were adjusted for the matching variables (age at survey return and time from diagnosis to survey return), and body mass index (BMI; calculated as weight in kilograms divided by height in meters squared). Additional adjusted models for factors associated with the PROMIS physical health score were also conducted. All variables included for each model were from the same survey in each time point. Stratified analyses by age (<65 and ≥65 years) at physical health assessment were conducted, and a likelihood ratio test *P* value was computed to evaluate a multiplicative interaction. Additionally, we conducted analyses restricted to women with estrogen receptor–positive and localized tumors to minimize the potential influence of tumor characteristics on physical health. Sample sizes for other clinical subgroups were too small for a formal analysis.

**Figure.**
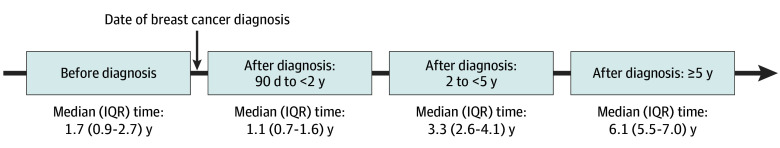
Physical Health Assessed at 4 Times Relative to the Diagnosis Date Median time refers to median time of physical health assessment relative to the diagnosis date. Women without cancer were matched to breast cancer survivors based on age and year of survey return.

Multivariable ordinal logistic regression models were used to compute odds ratios (ORs) and 95% CIs for the associations of treatment received with self-reported physical health, physical function, fatigue, and pain, the individual components used to compute the PROMIS physical health score. These models were also adjusted for the matching variables and BMI. Finally, associations of treatment received that included endocrine therapy types with the PROMIS physical health score were computed using linear regression as described above.

Analyses were conducted using the R software version 4.2.3 (R Project for Statistical Computing). All statistical tests were 2-sided; *P* < .05 was considered statistically significant.

## Results

This analysis included 15 392 women: 2566 women diagnosed with BC and 12 826 age-matched women without cancer. The study population included 184 Asian (1.2%), 418 Black (2.7%), 731 Hispanic (all races) (4.8%), and 13 758 White (89.4%) women (Table 1). Median (IQR) age at the prediagnosis physical health assessment was 54.1 (47.8-59.9) years for women diagnosed with BC. Women diagnosed with BC were more likely to be have a BMI of 30 or greater (750 women [29.2%]) compared with women without cancer (3376 women [26.3%]). Other characteristics were similar between groups. For the women diagnosed with BC, median (IQR) age at diagnosis was 56.3 (49.9-61.9) years (eTable 1 in Supplement 2). Most women were diagnosed after 2015 (1375 women [53.6%]) with tumors that were hormone receptor positive (2109 women [82.2%]), localized (1405 women [54.8%]), or in situ (608 women [23.7%]). There were 433 women with BC (16.9%) who did not receive chemotherapy nor endocrine therapy, 1223 women with BC (47.7%) who received endocrine therapy without chemotherapy, 276 women with BC (10.8%) who received chemotherapy without endocrine therapy, and 634 women with BC (24.7%) who received chemotherapy and endocrine therapy. Of 1857 women (72.4%) who received endocrine therapy, 591 (31.8%) used tamoxifen, and 888 (47.8%) used AIs (eTable 1 in Supplement 2).

**Table 1.** Characteristics Before Diagnosis for Women With BC (Overall and by Treatment Received) and Matched Survey for Women Without Cancer

Characteristics	Women, No. (%)					
	Without BC (n = 12 826)	With BC				
		Total (n = 2566)	No chemotherapy or endocrine therapy (n = 433)	Endocrine therapy (n = 1223)	Chemotherapy (n = 276)	Chemotherapy and endocrine therapy (n = 634)
Age, median (IQR), y	54.1 (47.8-59.9)	54.1 (47.7-60.0)	53.2 (46.9-60.0)	55.4 (49.2-61.1)	53.2 (45.9-58.8)	52.3 (45.7-57.8)
Race and ethnicity						
Hispanic (all races)	614 (4.8)	117 (4.6)	23 (5.3)	41 (3.4)	16 (5.8)	37 (5.8)
Non-Hispanic						
American Indian and Alaska Native	126 (1.0)	22 (0.9)	4 (0.9)	11 (0.9)	2 (0.7)	5 (0.8)
Asian	146 (1.1)	38 (1.5)	10 (2.3)	20 (1.6)	−2 (0.7)	6 (0.9)
Black	356 (2.8)	62 (2.4)	11 (2.5)	26 (2.1)	15 (5.4)	10 (1.6)
Native Hawaiian or Pacific Islander	10 (0.1)	2 (0.1)	0	1 (0.1)	0	1 (0.2)
White	11 461 (89.4)	2297 (89.5)	378 (87.3)	1113 (91.0)	240 (87.0)	566 (89.3)
Other^a^	21 (0.2)	6 (0.2)	0	2 (0.2)	0	4 (0.6)
Unknown/missing	92 (0.7)	22 (0.9)	7 (1.6)	9 (0.7)	1 (0.4)	5 (0.8)
Highest level of education						
≤High school	839 (6.5)	180 (7.0)	28 (6.5)	89 (7.3)	20 (7.2)	43 (6.8)
Some college	1534 (12.0)	285 (11.1)	41 (9.5)	119 (9.7)	38 (13.8)	87 (13.7)
2-y college/technical school	1521 (11.9)	292 (11.4)	40 (9.2)	146 (11.9)	31 (11.2)	75 (11.8)
4-y college degree	3442 (26.8)	675 (26.3)	113 (26.1)	325 (26.6)	69 (25.0)	168 (26.5)
Graduate degree	3008 (23.5)	594 (23.2)	110 (25.4)	293 (24.0)	62 (22.5)	129 (20.4)
Unknown/missing	2482 (19.4)	540 (21.0)	101 (23.3)	251 (20.5)	56 (20.3)	132 (20.8)
Income level, $						
<15 000	214 (1.7)	40 (1.6)	6 (1.4)	19 (1.6)	5 (1.8)	10 (1.6)
15 000-24 999	432 (3.4)	75 (2.9)	11 (2.5)	28 (2.3)	16 (5.8)	20 (3.2)
25 000-49 999	1841 (14.4)	344 (13.4)	57 (13.2)	169 (13.8)	35 (12.7)	83 (13.1)
50 000-99 999	4844 (37.8)	956 (37.3)	145 (33.5)	461 (37.7)	112 (40.6)	238 (37.5)
100 000-149 999	3001 (23.4)	631 (24.6)	118 (27.3)	283 (23.1)	66 (23.9)	164 (25.9)
≥150 000	2171 (16.9)	453 (17.7)	83 (19.2)	227 (18.6)	38 (13.8)	105 (16.6)
Unknown/missing	323 (2.5)	67 (2.6)	13 (3.0)	36 (2.9)	4 (1.5)	14 (2.2)
Health insurance status						
No	491 (3.8)	92 (3.6)	11 (2.5)	40 (3.3)	14 (5.1)	27 (4.3)
Yes	12 237 (95.4)	2457 (95.8)	417 (96.3)	1174 (96.0)	260 (94.2)	606 (95.6)
Unknown/missing	98 (0.8)	17 (0.7)	5 (1.2)	9 (0.7)	2 (0.7)	1 (0.2)
Menopausal status						
Premenopausal	4220 (32.9)	890 (34.7)	160 (37.0)	379 (31.0)	98 (35.5)	253 (39.9)
Postmenopausal	6450 (50.3)	1239 (48.3)	191 (44.1)	637 (52.1)	128 (46.4)	283 (44.6)
Unknown/missing	2156 (16.8)	437 (17.0)	82 (18.9)	207 (16.9)	50 (18.1)	98 (15.5)
Smoking						
Never	8733 (68.1)	1716 (66.9)	296 (68.4)	822 (67.2)	184 (66.7)	414 (65.3)
Current	421 (3.3)	81 (3.2)	14 (3.2)	30 (2.5)	9 (3.3)	28 (4.4)
Former	3636 (28.4)	761 (29.7)	122 (28.2)	366 (29.9)	83 (30.1)	190 (30.0)
Unknown/missing	36 (0.3)	8 (0.3)	1 (0.2)	5 (0.4)	0	2 (0.3)
Drinks per day						
Not a drinker	2336 (18.2)	412 (16.1)	60 (13.9)	199 (16.3)	48 (17.4)	105 (16.6)
<1	6752 (52.6)	1384 (53.9)	226 (52.2)	653 (53.4)	163 (59.1)	342 (53.9)
1-2	1193 (9.3)	241 (9.4)	43 (9.9)	130 (10.6)	20 (7.3)	48 (7.6)
≥3	434 (3.4)	90 (3.5)	19 (4.4)	45 (3.7)	5 (1.8)	21 (3.3)
Unknown/missing	2111 (16.5)	439 (17.1)	85 (19.6)	196 (16.0)	40 (14.5)	118 (18.6)
BMI						
<25	5591 (43.6)	996 (38.8)	220 (50.8)	458 (37.5)	95 (34.4)	223 (35.2)
25 to <30	3684 (28.7)	799 (31.1)	103 (23.8)	401 (32.8)	97 (35.1)	198 (31.2)
≥30	3375 (26.3)	750 (29.2)	108 (24.9)	358 (29.3)	81 (29.4)	203 (32.0)
Unknown/missing	176 (1.4)	21 (0.8)	2 (0.5)	6 (0.5)	3 (1.1)	10 (1.6)
Diabetes						
No	12 195 (95.1)	2407 (93.8)	416 (96.1)	1150 (94.0)	261 (94.6)	580 (91.5)
Yes	631 (4.9)	159 (6.2)	17 (3.9)	73 (6.0)	15 (5.4)	54 (8.5)
Hypertension						
No	9798 (76.4)	1918 (74.8)	353 (81.5)	891 (72.9)	204 (73.9)	470 (74.1)
Yes	3028 (23.6)	648 (25.3)	80 (18.5)	332 (27.2)	72 (26.1)	164 (25.9)

Abbreviations: BC, breast cancer; BMI, body mass index (calculated as weight in kilograms divided by height in meters squared).

^a^ Participants who selected other race or ethnicity had the option of a write-in text box.

Physical health was assessed at 4 time points relative to the patient’s diagnosis date (Figure; eFigure 2 in Supplement 1). Median (IQR) time from assessment to diagnosis was 1.7 (0.9-2.7) years, and median (IQR) times from diagnosis to the assessments were 1.1 (0.7-1.6) years, 3.3 (2.6-4.1) years, and 6.1 (5.5-7.0) years). Clinical and personal characteristics of the participants with physical health assessments at less vs more than 5 years of follow-up were similar except for year of diagnosis (eTable 2 and eTable 3 in Supplement 2). There were 881 BC survivors (34.3%) with 2 postdiagnosis measures in different intervals.

Prior to the BC diagnosis or matched surveys for women without cancer, 8211 women (61.1%) reported having excellent or very good health (eTable 4 in Supplement 2). During the first 2 years after diagnosis, 617 women with BC (43.3%) reported having excellent or very good health, compared with 4271 women without cancer (59.5%). This percentage was lowest in BC survivors who received chemotherapy (41 women [26.1%]) or chemotherapy and endocrine therapy (126 women [35.5%]). Five years after diagnosis, 1455 women without cancer (59.4%) and 126 women with BC who received endocrine therapy without chemotherapy (59.4%) reported having excellent or very good health. However, only 23 women who received chemotherapy without endocrine therapy (41.8%) and 66 women who received chemotherapy with endocrine therapy (50.0%) reported having excellent or very good health.

Compared with women without cancer, there was a greater physical health decline within 2 years of diagnosis for women with BC who received endocrine therapy (β = −1.12; 95% CI, −1.64 to −0.60 (Table 2), chemotherapy (β = −3.13; 95% CI, −4.19 to −2.07), or the combination of both (β = −3.26; 95% CI, −3.97 to −2.55). More than 2 years after diagnosis, a significant decline was only present among those who received chemotherapy. Additionally, models adjusted for factors associated with greater physical health decline (smoking, alcohol, diabetes, and hypertension) yielded the same results (eTable 5 in Supplement 2).There was not a significant interaction between treatment received and age at physical health decline (eTable 6 in Supplement 2). In analyses restricted to women with estrogen receptor–positive tumors and localized stage, a greater physical declined was observed among women receiving endocrine therapy (β = −1.40; 95% CI, −2.04 to −0.75) (eTable 7 in Supplement 2) and chemotherapy plus endocrine therapy (β = −2.89; 95% CI, −3.92 to −1.86) within 2 years from diagnosis. More than 5 years after diagnosis, this decline was only observed in women receiving chemotherapy.

**Table 2.** Association Between Treatment Received and Physical Health Score

Treatment received	Before diagnosis		90 d to <2 y		2 y to <5 y		≥5 y	
	Women, No. (%)	β (95% CI)^a^	Women, No. (%)	β (95% CI)^a^	Women, No. (%)	β (95% CI)^a^	Women, No. (%)	β (95% CI)^a^
**All breast cancer**								
Matched women without cancer	4898 (83.4)	0 [Reference]	7119 (83.5)	0 [Reference]	7059 (83.5)	0 [Reference]	2429 (83.2)	0 [Reference]
No chemotherapy nor endocrine therapy	146 (2.5)	−0.10 (−1.15 to 0.95)	218 (2.6)	−1.10 (−2.00 to −0.21)	246 (2.9)	−0.70 (−1.56 to 0.16)	93 (3.2)	0.18 (−1.21 to 1.59)
Endocrine therapy	505 (8.6)	−0.19 (−0.77 to 0.40)	687 (8.1)	−1.12 (−1.64 to −0.60)	659 (7.8)	−0.40 (−0.94 to 0.14)	210 (7.2)	−0.11 (−1.07 to 0.85)
Chemotherapy	98 (1.7)	−0.65 (−1.93 to 0.62)	152 (1.8)	−3.13 (−4.19 to −2.07)	141 (1.7)	−1.20 (−2.32 to −0.07)	55 (1.9)	−4.09 (−5.91 to −2.27)
Chemotherapy and endocrine therapy	225 (3.8)	0.17 (−0.68 to 1.03)	354 (4.2)	−3.26 (−3.97 to −2.55)	348 (4.1)	−1.34 (−2.07 to −0.61)	132 (4.5)	−1.65 (−2.85 to −0.46)
**Invasive breast cancer**								
Matched women without cancer	3768 (83.4)	0 [Reference]	5442 (83.5)	0 [Reference]	5248 (83.5)	0 [Reference]	1836 (83.3)	0 [Reference]
No chemotherapy nor endocrine therapy	33 (0.7)	−0.40 (−2.58 to 1.78)	50 (0.8)	−1.70 (−3.54 to 0.14)	58 (0.9)	−1.04 (−2.77 to 0.69)	22 (1.0)	0.89 (−1.92 to 3.70)
Endocrine therapy	394 (8.7)	−0.36 (−1.03 to 0.30)	525 (8.1)	−1.38 (−1.98 to −0.79)	496 (7.9)	−0.53 (−1.14 to 0.09)	162 (7.4)	−0.40 (−1.48 to 0.67)
Chemotherapy	98 (2.2)	−0.58 (−1.86 to 0.70)	152 (2.3)	−3.18 (−4.24 to −2.11)	140 (2.2)	−1.19 (−2.31 to −0.06)	53 (2.4)	−4.10 (−5.93 to −2.28)
Chemotherapy and endocrine therapy	224 (5.0)	0.33 (−0.53 to 1.19)	351 (5.4)	−3.34 (−4.05 to −2.62)	342 (5.4)	−1.41 (−2.14 to −0.67)	132 (6.0)	−1.80 (−2.98 to −0.62)
**In situ breast cancer^b^**								
Matched women without cancer	1125 (83.4)	0 [Reference]	1658 (83.4)	0 [Reference]	1777 (83.5)	0 [Reference]	583 (83.1)	0 [Reference]
No chemotherapy nor endocrine therapy	113 (8.4)	−0.28 (−1.51 to 0.95)	168 (8.5)	−0.82 (−1.92 to 0.19)	188 (8.8)	−0.41 (−1.45 to 0.64)	71 (10.1)	0.31 (−1.47 to 2.10)
Endocrine therapy	111 (8.2)	0.50 (−0.74 to 1.74)	162 (8.2)	−0.22 (−1.30 to 0.85)	163 (7.7)	0.08 (−1.03 to 1.19)	48 (6.8)	0.92 (−1.21 to 3.05)

^a^ Linear regression models adjusted for age, time to or from diagnosis, and body mass index. All variables in the models were obtained from the same survey in the indicated interval.

^b^ Analyses were restricted to endocrine therapy only, since clinical guidelines do not recommend chemotherapy for women diagnosed with in situ cancer.

Associations between treatment received and the 4 items of the physical health score as the outcome were also investigated (Table 3). Self-reported physical health and fatigue were comparable to the results for the overall score. However, women who received endocrine therapy without chemotherapy reported having worse physical health in the 2- to 5-year period after diagnosis compared with women without cancer (OR, 1.33; 95% CI, 1.15-1.55). There was no difference in the ability to carry out ADL in patients who received endocrine therapy without chemotherapy and women without cancer during the study period.

**Table 3.** Association Between Treatment Received and the Self-Reported Physical Health Score

Treatment received	Before diagnosis		90 d to <2 y		2 to <5 y		≥5 y	
	Women, No. (%)	OR (95% CI)^a^	Women, No. (%)	OR (95% CI)^a^	Women, No. (%)	OR (95% CI)^a^	Women, No. (%)	OR (95% CI)^a^
**Physical health^b^**								
Matched women without cancer	11239 (83.6)	1 [Reference]	7172 (83.4)	1 [Reference]	7124 (83.5)	1 [Reference]	2448 (83.3)	1 [Reference]
No chemotherapy nor endocrine therapy	367 (2.7)	0.90 (0.74-1.10)	220 (2.6)	1.29 (1.00-1.66)	250 (2.9)	1.24 (0.98-1.57)	93 (3.2)	1.21 (0.81-1.80)
Endocrine therapy	1055 (7.8)	0.98 (0.87-1.10)	693 (8.1)	1.68 (1.45-1.94)	665 (7.8)	1.33 (1.15-1.55)	212 (7.2)	1.02 (0.79-1.33)
Chemotherapy	245 (1.8)	1.06 (0.84-1.34)	157 (1.8)	3.76 (2.79-5.07)	142 (1.7)	1.59 (1.17-2.17)	55 (1.9)	2.25 (1.38-3.67)
Chemotherapy and endocrine therapy	546 (4.1)	1.04 (0.89-1.23)	355 (4.1)	2.66 (2.18-3.26)	349 (4.1)	1.64 (1.35-2.01)	132 (4.5)	1.74 (1.25-2.42)
**Ability to carry out everyday activities of daily living^c^**								
Matched women without cancer	4922 (83.4)	1 [Reference]	7172 (83.4)	1 [Reference]	7110 (83.5)	1 [Reference]	2452 (83.2)	1 [Reference]
No chemotherapy nor endocrine therapy	147 (2.5)	1.12 (0.73-1.68)	220 (2.6)	1.53 (1.12-2.06)	250 (2.9)	1.27 (0.94-1.70)	93 (3.16)	1.22 (0.73-1.98)
Endocrine therapy	507 (8.6)	0.93 (0.73-1.17)	693 (8.1)	1.12 (0.93-1.34)	665 (7.8)	0.99 (0.82-1.20)	215 (7.3)	1.07 (0.76-1.49)
Chemotherapy	98 (1.7)	1.22 (0.74-1.93)	156 (1.8)	1.99 (1.40-2.79)	141 (1.7)	1.34 (0.91-1.93)	55 (1.9)	2.49 (1.42-4.23)
Chemotherapy and endocrine therapy	225 (3.8)	0.91 (0.64-1.26)	356 (4.1)	2.30 (1.84-2.87)	350 (4.1)	1.35 (1.05-1.72)	132 (4.5)	1.39 (0.93-2.03)
**Fatigue^d^**								
Matched women without cancer	4919 (83.4)	1 [Reference]	7168 (83.5)	1 [Reference]	7117 (83.5)	1 [Reference]	2451 (83.3)	1 [Reference]
No chemotherapy nor endocrine therapy	147 (2.5)	1.09 (0.80-1.49)	218 (2.5)	1.24 (0.95-1.60)	249 (2.9)	1.18 (0.93-1.49)	93 (3.2)	0.70 (0.46-1.04)
Endocrine therapy	507 (8.6)	1.06 (0.89-1.26)	693 (8.1)	1.22 (1.05-1.41)	666 (7.8)	1.12 (0.96-1.30)	213 (7.2)	0.99 (0.75-1.29)
Chemotherapy	98 (1.7)	1.25 (0.85-1.84)	153 (1.8)	1.71 (1.25-2.35)	142 (1.7)	1.45 (1.05-1.99)	55 (1.9)	2.78 (1.68-4.60)
Chemotherapy and endocrine therapy	225 (3.8)	1.03 (0.80-1.33)	356 (4.2)	1.95 (1.59-2.39)	349 (4.1)	1.29 (1.05-1.58)	132 (4.5)	1.33 (0.96-1.85)
**Pain^e^**								
Matched women without cancer	4924 (83.4)	1 [Reference]	7177 (83.5)	1 [Reference]	7121 (83.5)	1 [Reference]	2446 (83.2)	1 [Reference]
No chemotherapy nor endocrine therapy	147 (2.5)	1.06 (0.77-1.47)	219 (2.6)	1.12 (0.86-1.45)	250 (2.9)	0.97 (0.76-1.24)	93 (3.2)	0.93 (0.62-1.39)
Endocrine therapy	508 (8.6)	1.07 (0.90-1.29)	693 (8.1)	0.99 (0.86-1.16)	666 (7.8)	0.93 (0.79-1.08)	215 (7.3)	1.01 (0.77-1.33)
Chemotherapy	98 (1.7)	0.99 (0.67-1.45)	156 (1.8)	1.16 (0.85-1.58)	142 (1.7)	0.98 (0.72-1.35)	55 (1.9)	1.94 (1.12-3.33)
Chemotherapy and endocrine therapy	225 (3.8)	1.00 (0.77-1.30)	355 (4.1)	1.25 (1.02-1.54)	350 (4.1)	1.07 (0.87-1.32)	132 (4.5)	1.10 (0.78-1.54)

Abbreviation: OR, odds ratio.

^a^ Ordinal logistic regression models adjusted for age, time to or from diagnosis, and body mass index. All variables in the models were obtained from the same survey in the indicated interval.

^b^ Physcial health was coded with higher values indicated poor health, and lower values indicating excellent health.

^c^ Ability to carry out everyday activities of daily living (walking, climbing stairs, carrying groceries, or moving a chair) was coded with higher values indicating inability and lower values indicating complete ability.

^d^ Fatigue was coded with higher values indicated very severe fatigue, and lower values indicating no fatigue.

^e^ Pain was coded with higher values indicated worst pain, and lower values indicating no pain.

Lastly, we explored the association between type of endocrine therapy and physical health (Table 4). Compared with women without cancer, there was a greater physical health decline within 2 years of diagnosis for women who received AIs (β = −1.28; 95% CI, −2.06 to −0.49), chemotherapy (β = −3.13; 95% CI, −4.19 to −2.06), or both (β = −3.61; 95% CI, −4.58 to −2.64). However, more than 2 years after diagnosis, the greater decline was only observed in women who received chemotherapy (2 to 5 years: β = −1.20; 95% CI, −2.32 to −0.07; ≥5 years: β = −4.09; 95% CI, −5.91 to −2.27), or chemotherapy plus AIs (2 to 5 years: β = −1.43; 95% CI, −2.49 to −0.38; ≥5 years: β = −2.03; 95% CI, −3.81 to −0.26). Women receiving tamoxifen, with or without chemotherapy, did not experience physical health decline compared with age-matched women without cancer.

**Table 4.** Association Between Treatment Received With Types of Endocrine Therapy and Physical Health Score

Treatment received	Before diagnosis		90 d to <2 y		2 to <5 y			≥5 y		
	Women, No. (%)	β (95% CI)^a^	Women, No. (%)	β (95% CI)^a^	Women, No. (%)	β (95% CI)^a^	Women, No. (%)		β (95% CI)^a^
Matched women without cancer	4898 (83.4)	0 [Reference]	7119 (83.5)	0 [Reference]	7059 (83.5)	0 [Reference]	2429 (83.2)		0 [Reference]
No chemotherapy nor endocrine therapy	146 (2.5)	−0.10 (−1.15 to 0.95)	218 (2.6)	−1.10 (−1.99 to −0.21)	246 (2.9)	−0.70 (−1.56 to 0.16)	93 (3.2)		0.18 (−1.23 to 1.59)
Tamoxifen	120 (2.0)	−0.17 (−1.32 to 0.99)	187 (2.2)	−0.67 (−1.64 to 0.29)	204 (2.4)	0.17 (−0.77 to 1.11)	69 (2.4)		−0.38 (−2.02 to 1.25)
Aromatase inhibitors	220 (3.8)	0.28 (−0.59 to 1.14)	287 (3.4)	−1.28 (−2.06 to −0.49)	252 (3.0)	−0.40 (−1.25 to 0.45)	73 (2.5)		0.84 (−0.75 to 2.44)
Chemotherapy	98 (1.7)	−0.65 (−1.93 to 0.62)	152 (1.8)	−3.13 (−4.19 to −2.06)	141 (1.7)	−1.20 (−2.32 to −0.07)	55 (1.9)		−4.09 (−5.91 to −2.27)
Chemotherapy and tamoxifen	42 (0.7)	1.14 (−0.80 to 3.09)	68 (0.8)	−1.34 (−2.93 to 0.25)	79 (0.9)	−0.43 (−1.93 to 1.08)	36 (1.2)		−0.40 (−2.65 to 1.86)
Chemotherapy and aromatase inhibitors	107 (1.8)	0.15 (−1.07 to 1.37)	183 (2.2)	−3.61 (−4.58 to −2.64)	162 (1.9)	−1.43 (−2.49 to −0.38)	58 (2.0)		−2.03 (−3.81 to −0.26)

^a^ Linear regression models adjusted for age, time to or from diagnosis, and body mass index. All variables in the models were obtained from the same survey in the indicated interval.

## Discussion

In this large, contemporary cohort study of women with physical health measurements at multiple times, most women with BC experienced a greater decline in their physical health compared with age-matched women without cancer during the first 2 years from diagnosis. Patients receiving chemotherapy (with or without endocrine therapy) had the greatest overall physical health decline, and it was long-lasting. However, the physical health of women with BC who received endocrine therapy without chemotherapy was similar to age-matched women without cancer after 2 years from diagnosis, regardless of type of endocrine therapy received.

The short-term effects of chemotherapy on physical health in BC survivors have been previously reported in older adults.^11,26^ We also observed this decline in physical health in middle-aged adults. Most of these previous studies have focused on physical health in the year following the diagnosis and have observed pronounced declines. Guidelines have recommended endocrine therapy for at least 5 years since the early 2000s^14^; therefore, it is important to understand long-term effects of systemic treatment, especially for endocrine therapy. We observed that endocrine therapy was significantly associated with physical health decline in the first 2 years after diagnosis among women with BC but similar to age-matched women without cancer after that. Given the 5-year recommendation for endocrine therapy, it is possible that proper counseling and proactive management of the side effects of endocrine therapy were implemented for some women after the first years, resulting in improved physical health.^27,28^ Nonadherence to endocrine therapies due to the side effects is also well known,^29,30^ but we could not investigate this in our study.

Endocrine therapy, and especially tamoxifen, has been associated with lower incidence of certain age-related conditions^31^ compared with other treatment, including cardiovascular disease,^32,33^ osteoporosis and osteopenia,^34^ and cognitive decline.^35,36^ We investigated the associations of tamoxifen and AIs with physical health and observed that women using tamoxifen with or without chemotherapy had physical health similar to age-matched women without cancer. Women receiving AIs without chemotherapy experienced a decline in physical health within the first 2 years of diagnosis, but their physical health was similar to women without cancer after 2 years. These results are in agreement with a prior small study (60 women receiving AIs and 66 receiving tamoxifen) that examined the associations of AIs with physical health at 6 and 12 months after diagnosis and found that women using AIs also had worse physical health compared with women using tamoxifen.^37^ Common adverse effects of AIs are musculoskeletal and bone pains,^38,39^ which may explain the greater physical health decline among these women. As treatment patterns change over time^40,41,42^ and AIs may become the main form of treatment for estrogen-receptor positive BC,^43^ survivorship care must be carefully reevaluated to understand the needs of these women.

Finally, we explored physical health–related factors associated with the physical health decline. We observed that increased fatigue and reduced ability to carry out ADL (eg, walking, climbing stairs, or carrying groceries) were the most pronounced factors of physical health decline among women with BC who received chemotherapy (with or without endocrine therapy). However, among women who received endocrine therapy alone, the physical deficits were limited to lower self-reported physical health and increased fatigue.

### Limitations

The findings from this analysis should be considered with certain limitations. Treatment information was limited to first-line treatment, and we did not have information on the types of chemotherapy received, which could influence physical health differently. Similarly, we did not have information on other treatments, such as targeted therapies or immunotherapies, which also could have influenced physical health. For some specific treatments, numbers were small, and so we may have had limited statistical power to observe associations. New guidelines recommend adjuvant endocrine therapy for up to 10 years.^44^ We currently do not have enough extended follow-up to investigate the physical health decline related to endocrine therapy beyond this recommended time. There were 228 women with BC (8.9%) who did not return a second survey, limiting the ability to assess longer-term physical health for these women. If failure to return the survey was not random but instead due to poor physical health, we may have seen stronger associations had they returned their survey. Future surveys may be able to provide information about long-term physical health among these women. Furthermore, findings from our analysis may not be generalizable to all women receiving treatment for BC, given that our sample mostly included non-Hispanic White women who had higher education and income than the national averages. Studies that include more diverse populations are needed.

## Conclusions

Our cohort study found a greater physical health decline in women receiving chemotherapy, with or without endocrine therapy, which persisted more than 5 years after the diagnosis compared with age-matched women without cancer. In contrast, the physical health decline observed in women who received endocrine therapy without chemotherapy was restricted to the first 2 years after diagnosis. Breast cancer is the most prevalent cancer among cancer survivors, accounting for more than 20% of all cancer survivors.^2^ Understanding the survivorship care needs and management of late effects in BC survivors is critical. Continued efforts are needed to understand the specific survivorship care needs of BC survivors and improve their long-term quality of life.

## Notes

Supplement 1. **eFigure 1.** Diagram of the Exclusion Criteria of Study Participants **eFigure 2.** Participants Included in Each Interval

Supplement 2. **eTable 1.** Clinical Characteristics of the Women With Breast Cancer **eTable 2.** Characteristics the Women With BC and Matched Survey for the Cancer-Free Women With Less or More Than 5 Years of Follow-Up **eTable 3.** Clinical Characteristics of the Women With BC With Less and More Than 5 Years of Follow-Up **eTable 4.** Descriptive Statistics of the PROMIS Physical Health Score and the Individual Items Included in the Score **eTable 5.** Association Between Treatment Received and the PROMIS Physical Health Score With Additionally Adjusted Variables **eTable 6.** Association Between Treatment Received and the PROMIS Physical Health Score, Stratified by Age at Physical Health Assessment **eTable 7.** Association Between Treatment Received and the PROMIS Physical Health Score, Restricted to Women With Estrogen Receptor–Positive and Localized Disease and Matched Cancer-Free Women

Supplement 3.
Data Sharing Statement
