# Supplementary material for: Physical Health Decline After Chemotherapy or Endocrine Therapy in Breast Cancer Survivors
Source: JAMA Netw Open. 2025 Feb 28;8(2):e2462365. doi: 10.1001/jamanetworkopen.2024.62365 (PMC11871543; doi:10.1001/jamanetworkopen.2024.62365)
Supplement: Supplement 1. — eFigure 1. Diagram of the Exclusion Criteria of Study Participants eFigure 2. Participants Included in Each Interval [file jamanetwopen-e2462365-s001.pdf]

Supplemental Online Content

Bodelon C, Masters M, Bloodworth DE, et al. Physical health decline after chemotherapy or endocrine therapy in breast cancer survivors. *JAMA Netw Open*. 2025;8(2):e2462365. doi:10.1001/jamanetworkopen.2024.62365

eFigure 1. Diagram of the Exclusion Criteria of Study Participants

eFigure 2. Participants Included in Each Interval

This supplemental material has been provided by the authors to give readers additional information about their work.

© 2025 Bodelon C et al. *JAMA Network Open*.

eFigure 1. Diagram of the exclusion criteria of study participants.

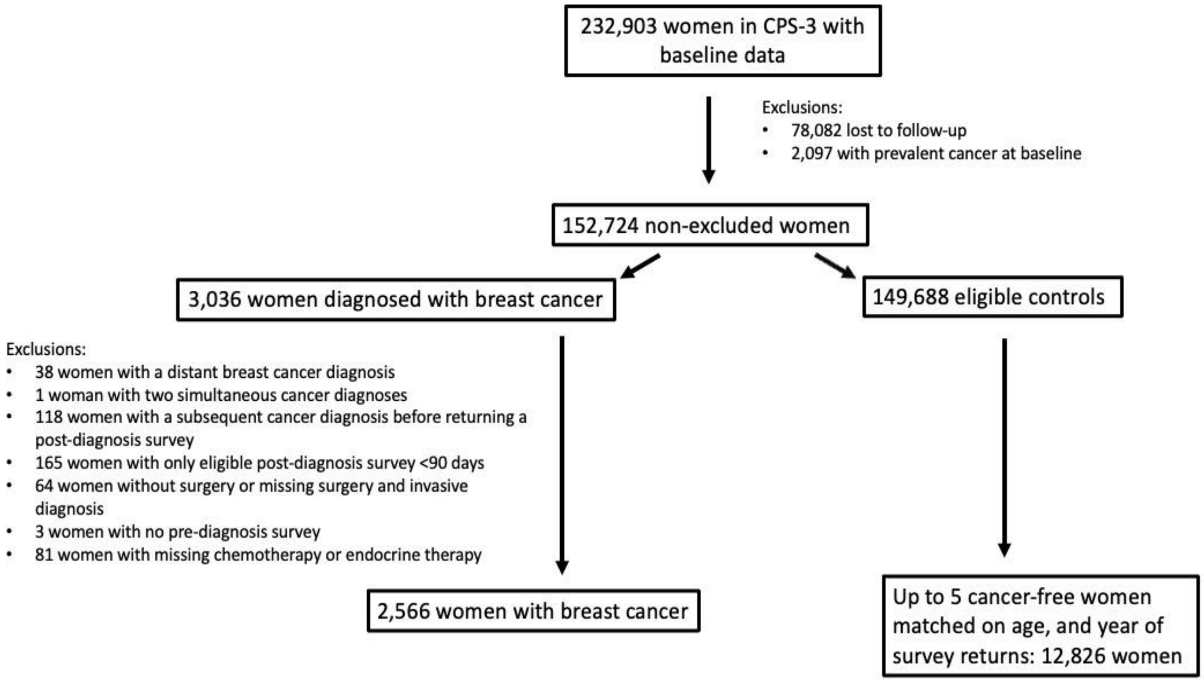

© 2025 Bodelon C et al. *JAMA Network Open*.

eFigure 2. Participants included in each interval.

Number of women with breast cancer and age-matched cancer-free with available outcome data at each interval*

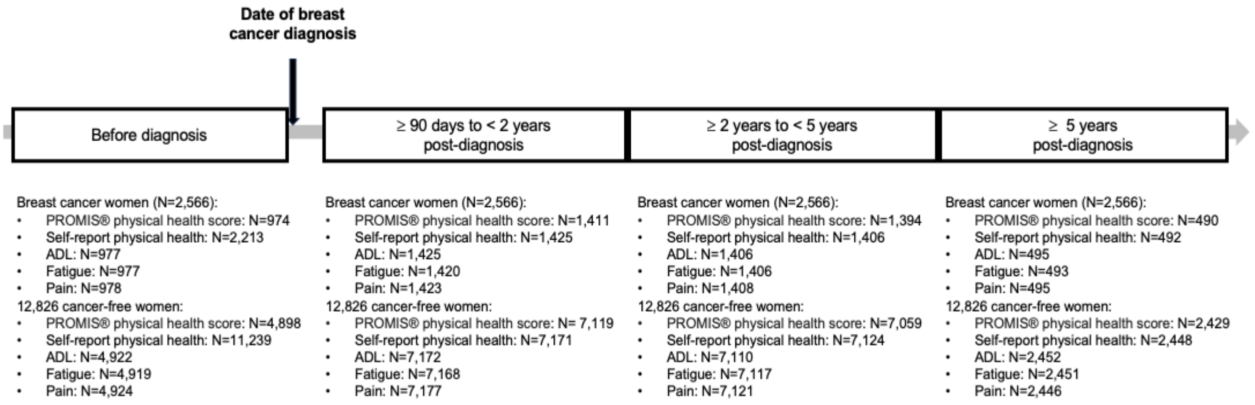

*Self-report physical health was available in the baseline, 2015, and 2018 surveys.
Ability to carry out activities of daily living (ADL), fatigue and pain was available in the 2015 and 2018 surveys.
PROMIS® physical health score could only be computed with data from the 2015 and 2018 surveys.

© 2025 Bodelon C et al. *JAMA Network Open*.
