# Supplementary material for: Physical Health Decline After Chemotherapy or Endocrine Therapy in Breast Cancer Survivors
Source: JAMA Netw Open. 2025 Feb 28;8(2):e2462365. doi: 10.1001/jamanetworkopen.2024.62365 (PMC11871543; doi:10.1001/jamanetworkopen.2024.62365)
Supplement: Supplement 3. — Data Sharing Statement [file jamanetwopen-e2462365-s003.pdf]

Data Sharing Statement

Bodelon. Physical Health Decline After Chemotherapy or Endocrine Therapy in Breast Cancer Survivors. *JAMA Netw Open*. Published February 28, 2025.
doi:10.1001/jamanetworkopen.2024.62365

Data
Data available: Yes
Data types: Deidentified participant data, Data dictionary
How to access data: Data are available from the American Cancer Society (ACS) by following the ACS Data Access Procedures (https://www.cancer.org/content/dam/cancer-org/research/epidemiology/cancer-prevention-study-data-access-policies.pdf) for researchers who meet the criteria for access to confidential data. Please email cohort.data@cancer.org to inquire about access.
When available: With publication

Supporting Documents
Document types: None

Additional Information
Who can access the data: Researchers whose proposed use of the data has been approved as indicated above
Types of analyses: For an approved proposal.
Mechanisms of data availability: With investigator support, after approval of a proposal and a signed data access agreement.
